# *In Silico* Investigation of Traditional Chinese Medicine for Potential Lead Compounds as SPG7 Inhibitors against Coronary Artery Disease

**DOI:** 10.3390/molecules21050588

**Published:** 2016-05-05

**Authors:** Kuen-Bao Chen, Kuan-Chung Chen, Ya-Lin Chang, Kun-Lung Chang, Pei-Chun Chang, Tung-Ti Chang, Yu-Chian Chen

**Affiliations:** 1 Department of Bioinformatics and Medical Engineering, Asia University, Taichung 41354, Taiwan; d3510@mail.cmuh.org.tw (K.-B.C.); midwives0336@yahoo.com.tw (Y.-L.C.); kuen90@yahoo.com.tw (K.-L.C.); peichun.chang@gmail.com (P.-C.C.); 2 School of Medicine, College of Medicine, China Medical University, Taichung 40402, Taiwan; 3 Department of Anesthesiology, China Medical University Hospital, Taichung 40447, Taiwan; 4 School of Pharmacy, China Medical University, Taichung 40402, Taiwan; 9818chen@gmail.com; 5 Department of Pharmacy, China Medical University Hospital, China Medical University, Taichung 40402, Taiwan; 6 School of Post-Baccalaureate Chinese Medicine, College of Chinese Medicine, China Medical University, Taichung 40402, Taiwan; 7 Department of Chinese Pediatrics, China Medical University Hospital, Taichung 40402, Taiwan; 8 Research Center for Chinese Medicine and Acupuncture, China Medical University, Taichung 40402, Taiwan; 9 Department of Medical Research, China Medical University Hospital, Taichung 40447, Taiwan; 10 Computational and Systems Biology, Massachusetts Institute of Technology, Cambridge, MA 02139, USA

**Keywords:** spastic paraplegia 7 (SPG7), Traditional Chinese Medicine (TCM), docking, molecular dynamics (MD) simulation

## Abstract

Coronary artery disease (CAD) is the most common cause of heart attack and the leading cause of mortality in the world. It is associated with mitochondrial dysfunction and increased level of reactive oxygen species production. According to the Ottawa Heart Genomics Study genome-wide association study, a recent research identified that Q688 spastic paraplegia 7 (SPG7) variant is associated with CAD as it bypasses the regulation of tyrosine phosphorylation of AFG3L2 and enhances the processing and maturation of SPG7 protein. This study aims to identify potential compounds isolated from Traditional Chinese Medicines (TCMs) as potential lead compounds for paraplegin (SPG7) inhibitors. For the crystallographic structure of paraplegin, the disordered disposition of key amino acids in the binding site was predicted using the PONDR-Fit protocol before virtual screening. The TCM compounds saussureamine C and 3-(2-carboxyphenyl)-4(3*H*)-quinazolinone, have potential binding affinities with stable H-bonds and hydrophobic contacts with key residues of paraplegin. A molecular dynamics simulation was performed to validate the stability of the interactions between each candidate and paraplegin under dynamic conditions. Hence, we propose these compounds as potential candidates as lead drug from the compounds isolated from TCM for further study in drug development process with paraplegin protein for coronary artery disease.

## 1. Introduction

Coronary artery disease (CAD), which can also be called coronary atherosclerotic heart disease or ischemic heart disease, is not only the most common cause of heart attack, but also one of the leading causes of mortality in the world [1,2,3,4]. When the inner layer of a coronary artery is damaged, plaques will build up at the site to repair the damage. The formation of multiple atheromatous plaques cause an artery wall thickens in a process called atherosclerosis [5]. The narrow arteries will then restrict blood flow to the heart, increasing the risk of heart attack. Many studies indicate that mitochondrial dysfunction and increased reactive oxygen species production levels are also associated with CAD [6,7,8,9,10,11]. Recently, many studies aim to identify the pathogenesis of diseases in order to determine the potential target proteins for drug design [12,13,14]. According to the Ottawa Heart Genomics Study (OHGS) genome-wide association study (GWAS), recent researches identified that Q688 spastic paraplegia 7 (SPG7) variant is associated with CAD and migraine. Q688 SPG7 variant bypass the regulation of tyrosine phosphorylation of AFG3L2 and enhance the processing and maturation of SPG7 protein and ATP production [15,16].

Human paraplegin (SPG7), which is a member of the ATPases associated with diverse cellular activities (AAA) protein family, is a nuclear-encoded mitochondrial metalloprotease protein [17,18]. It consists of a FtsH extracellular domain, AAA-domain, and metallopeptidase M41 domain [19,20].

In recent studies, compounds from Traditional Chinese Medicine (TCM) have been identified as potential lead candidates for the treatment of cancers, inflammation, and some other common diseases [21,22,23,24]. In the absence of specific evidence about the TCM targets and binding mode, a computational approach involving virtual screening and molecular dynamics (MD) simulations could pave the way for new insights into TCMs as promising therapeutic agents, as successfully described in literature about other case studies [25,26]. To develop a systematic investigation of TCMs, we employed the TCM compounds from the TCM Database@Taiwan [27] to identify potential lead compounds as paraplegin (SPG7) inhibitors. To discuss the structurally disordered disposition in the protein which may induce side-effects and reduce the binding affinity between ligand and target protein [28], we executed the PONDR-Fit protocol to predict the disordered disposition of paraplegin protein before virtual screening. Moreover, a MD simulation was performed after virtual screening to validate the stability of interactions between the paraplegin protein and each potential candidate.

## 2. Materials and Methods

### 2.1. Data Collection

The X-ray crystallography structure of the human AAA+ protein paraplegin (SPG7) downloaded from RCSB Protein Data Bank with PDB ID: 2QZ4 [20] was employed as the target protein. The sequence of the paraplegin protein from Swiss-Prot (UniProtKB: Q9UQ90) was employed to predict the disordered amino acids using the PONDR-Fit [29] protocol. The X-ray crystallography structure of paraplegin protein was prepared by the Prepare Protein module in Discovery Studio 2.5 (DS 2.5) to protonate the amino acids and removed crystal water in the X-ray crystallography structure. The TCM compounds were obtained from TCM Database@Taiwan [27], and each compound was prepared by Prepare Ligand module in DS 2.5 to protonate the molecular structure and then filtered using Lipinski’s Rule of Five [30].

### 2.2. Docking Simulation

The LigandFit protocol [31] in DS 2.5 was employed to virtually screen the TCM compounds and obtain the docking pose in the binding site using a shape filter and Monte-Carlo ligand conformation generation. The suitable docking poses were minimized using the Chemistry at HARvard Macromolecular Mechanics (CHARMM) force field [32] and similar poses were filtered using the clustering algorithm. In this study, we ranked the candidates using the scoring function of Dock Score [31] and considering hydrogen bonds (H-bonds) with residues of paraplegin.

### 2.3. Molecular Dynamics Simulation

Gromacs 4.5.5 [33] was employed to perform the molecular dynamics (MD) simulation. The topology and parameters of paraplegin protein and each candidate were obtained by preparation of pdb2gmx protocol of Gromacs and SwissParam program [34], respectively. The protocol in Gromacs created a cubic box based upon the edge approx 1.2 nm from the protein complexes periphery and solvated with TIP3P water model and 0.145 M NaCl model to form a neutral system. Steepest descents [35] minimization with a maximum of 5000 steps was performed to remove bad van der Waals contacts in the minimization section. Then the protocol in Gromacs performed a position-restrained molecular dynamics with the Linear Constraint algorithm for all bonds in the equilibration section with the condition of NVT equilibration, Berendsen weak thermal coupling method, and Particle Mesh Ewald method. The protocol in Gromacs performed 20 ns trajectories with time step in unit of 2 fs in production simulation with the condition of NPT ensembles and Particle Mesh Ewald (PME) option.

A series of protocols in Gromacs were employed in the MD trajectories analysis. The g_rms protocol was performed to calculate the root mean square deviation (RMSD) [36] to observe the variation of overall structure during the dynamic simulation process. The g_gyrate protocol was performed to measure the radius of gyration of atomic groups as a function of time to observe the variation of compactness during the dynamic simulation process. The g_msd protocol was performed to mean square displacement to observe the diffusion during the dynamic simulation process. The g_energy protocol was performed to analyze the variation of total energy during the dynamic simulation process. The g_rmsf protocol was performed to analyze the root mean square fluctuation (RMSF) of each amino acid to determine the flexibility during the dynamic simulation process. The g_covar and g_anaeig protocols were performed for principal components analysis to analyze the eigenvectors. In addition, this study also performed the protocols to analyze the variation of distance of hydrogen bonds, secondary structure, and calculate the minimum distances between amino acid residues.

## 3. Results and Discussion

### 3.1. Disordered Prediction

**Figure 1:**
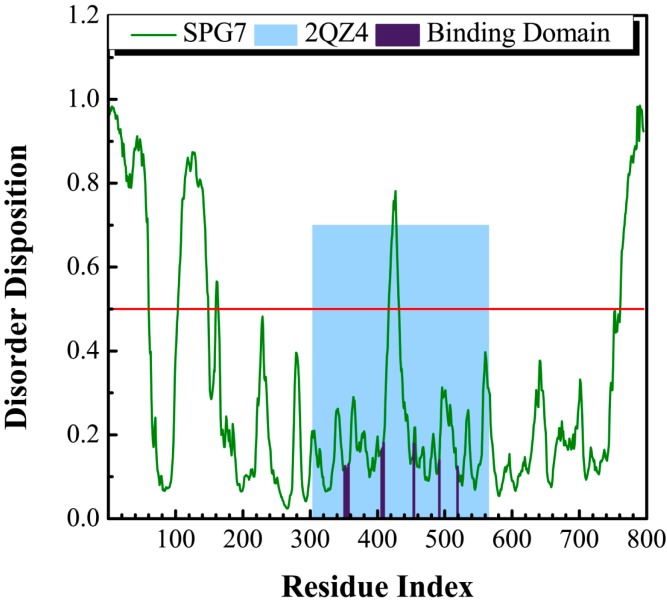
Disordered disposition for amino acids of paraplegin predicted by PONDR-Fit.

The sequence of paraplegin protein from Swiss-Prot (UniProtKB: Q9UQ90) was employed to predict the disordered disposition for each amino acids of paraplegin using the PONDR-Fit protocol. In Figure 1, all the key amino acids in the binding site in protein folding do not lay in a disordered domain (>0.5).

This indicates that the structure of the binding domain was folding stably with these amino acids and expresses a stable binding site for docking the suitable compounds. As a result, the crystallographic structure of paraplegin was employed for virtual screening to identify the potential lead compounds.

### 3.2. Docking Simulation

**Figure 2:**
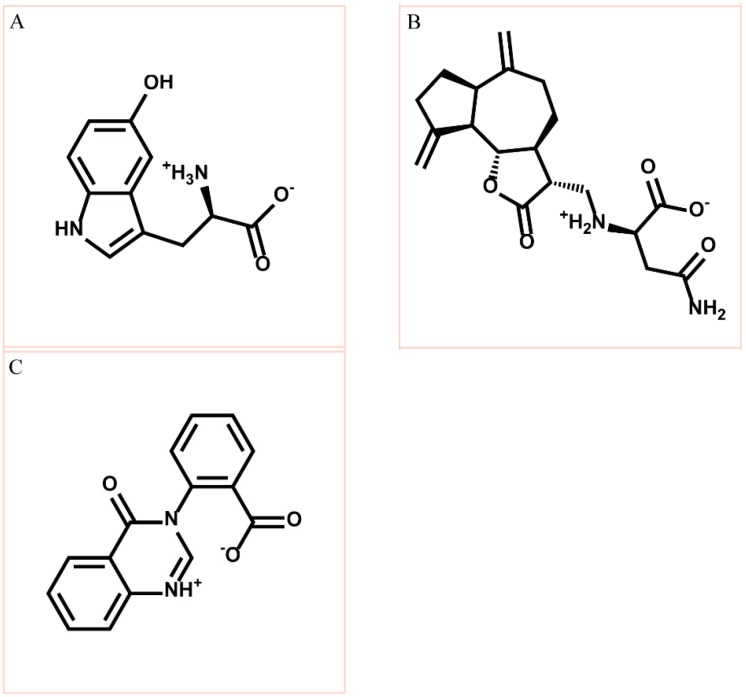
Chemical scaffold of top three TCM candidates, (**A**) 5-hydroxy-l-tryptophan; (**B**) saussureamine C; and (**C**) 3-(2-carboxyphenyl)-4(3*H*)-quinazolinone.

We employed the LigandFit protocol in DS 2.5 to virtually screen the TCM compounds and ranked the candidates using the scoring function of Dock Score and considering H-bonds with residues of paraplegin (Table 1). Figure 2 displayed the chemical scaffolds of the top three TCM candidates which have H-bonds with key residues of paraplegin and higher Dock score.

**Figure 3:**
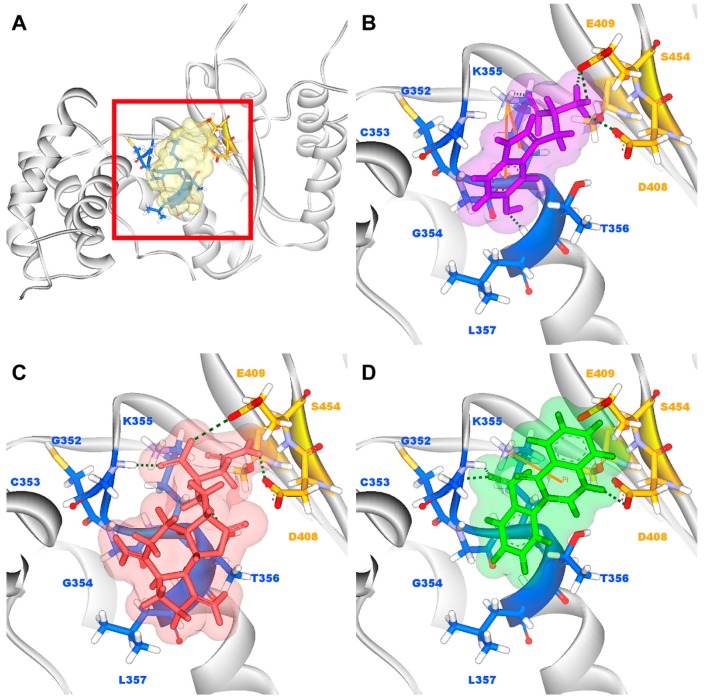
(**A**) Binding site of paraplegin and docking pose of paraplegin complexes with (**B**) 5-hydroxy-l-tryptophan; (**C**) saussureamine C; and (**D**) 3-(2-carboxyphenyl)-4(3*H*)-quinazolinone.

**Figure 4:**
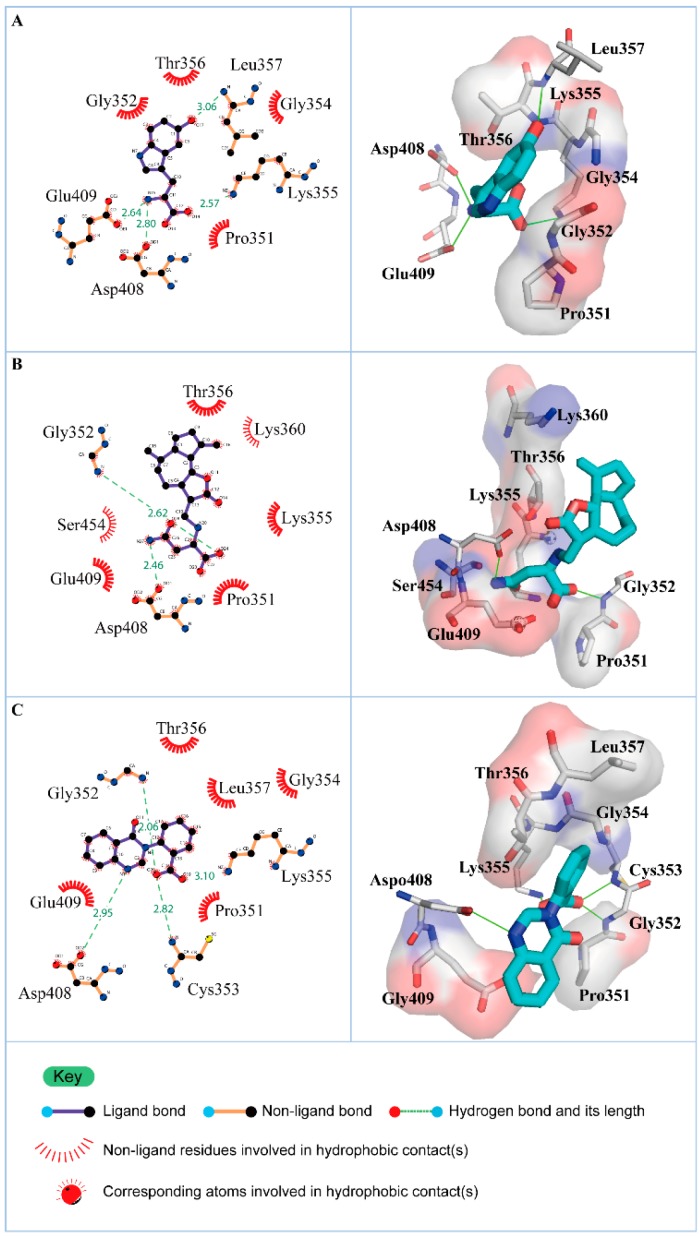
Hydrophobic contacts between residues of paraplegin and (**A**) 5-hydroxy-l-tryptophan; (**B**) saussureamine C; and (**C**) 3-(2-carboxyphenyl)-4(3*H*)-quinazolinone.

According to the information from TCM databases [27], 5-hydroxy-l-tryptophan is extracted from *Mucuna pruriens* seed, which has been indicated the function of antiproliferative effect [37], neuroprotective effect [38], control blood pressure [39]. In addition, 5-hydroxy-l-tryptophan is also the precursor to biosynthesis of 5-HT. Saussureamine C, extracted from *Saussurea lappa* Clarke, which has anti-ulcer principles [40], anti-oxidant activity [41], antihepatotoxic activity [42], and the function of ameliorate oxidative myocardial injury [43]. 3-(2-Carboxyphenyl)-4(3*H*)-quinazolinone is extracted from *Isatis indigotica* [44], which shows antiviral activity [45,46], antipyretic, antiviral, anti-inflammatory, anti-endotoxin activity, anticancer [47], and inhibitory effects on nitric oxide production [48]. Considering the interactions between each candidate and paraplegin in the binding domain shown in Figure 3A, the top candidates compounds have H-bonds with key residues in the chain from Gly352 to Thr356 (blue) and residues Asp408, Glu409, Ser454 (yellow) (Figure 3B–D), and hydrophobic contacts with residues Pro351, Gly352, Lys355, Thr356, Asp408, and Glu409 (Figure 4), which remain those compounds stable in the binding domain with similar docking poses. In the docking simulation result, the TCM candidates bind with the key residues of the α-helix (Pro351 to Lys360) and β-sheet (Asp408, Glu409, Ser454) in the binding domain of paraplegin. These interactions keep the compounds binding steady in the binding domain of paraplegin.

### 3.3. Molecular Dynamics Simulation

As a docking simulation performed by LigandFit protocol using a rigid body of paraplegin protein, the interactions between each candidates and paraplegin may not be stable under dynamic conditions. For this reason, the MD simulations were performed by Gromacs to validate the stability of interactions existed in the docking simulation.

**Figure 5:**
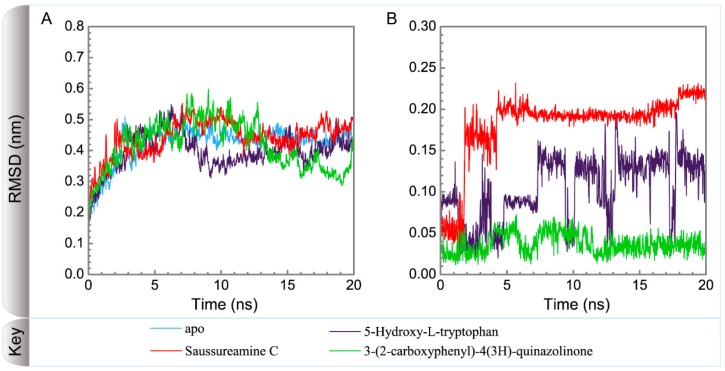
Variation of root-mean-square deviations, of (**A**) protein and (**B**) ligand over 20 ns for paraplegin in apo form and in complexes with three TCM candidates.

**Figure 6:**
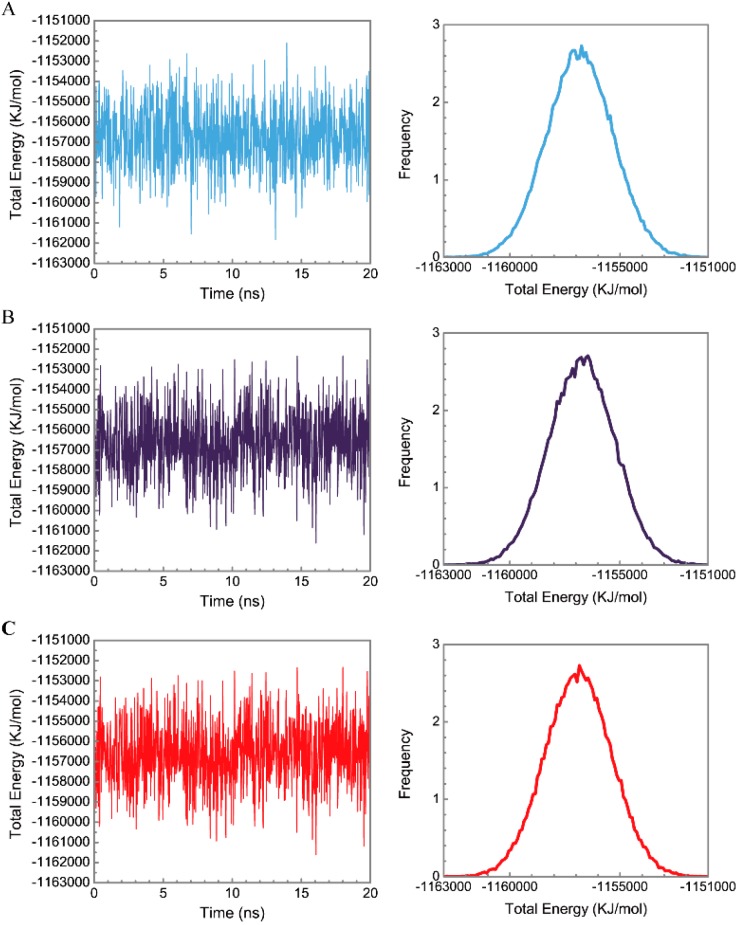

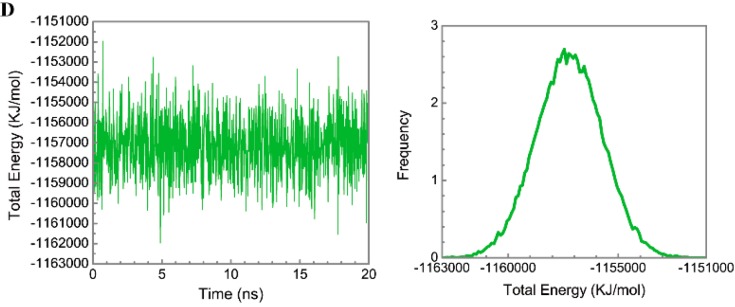
Distribution and variation of total energy for paraplegin protein in (**A**) apo form and complexes with (**B**) 5-hydroxy-l-tryptophan; (**C**) saussureamine C; and (**D**) 3-(2-carboxyphenyl)-4(3*H*)-quinazolinone.

**Figure 7:**
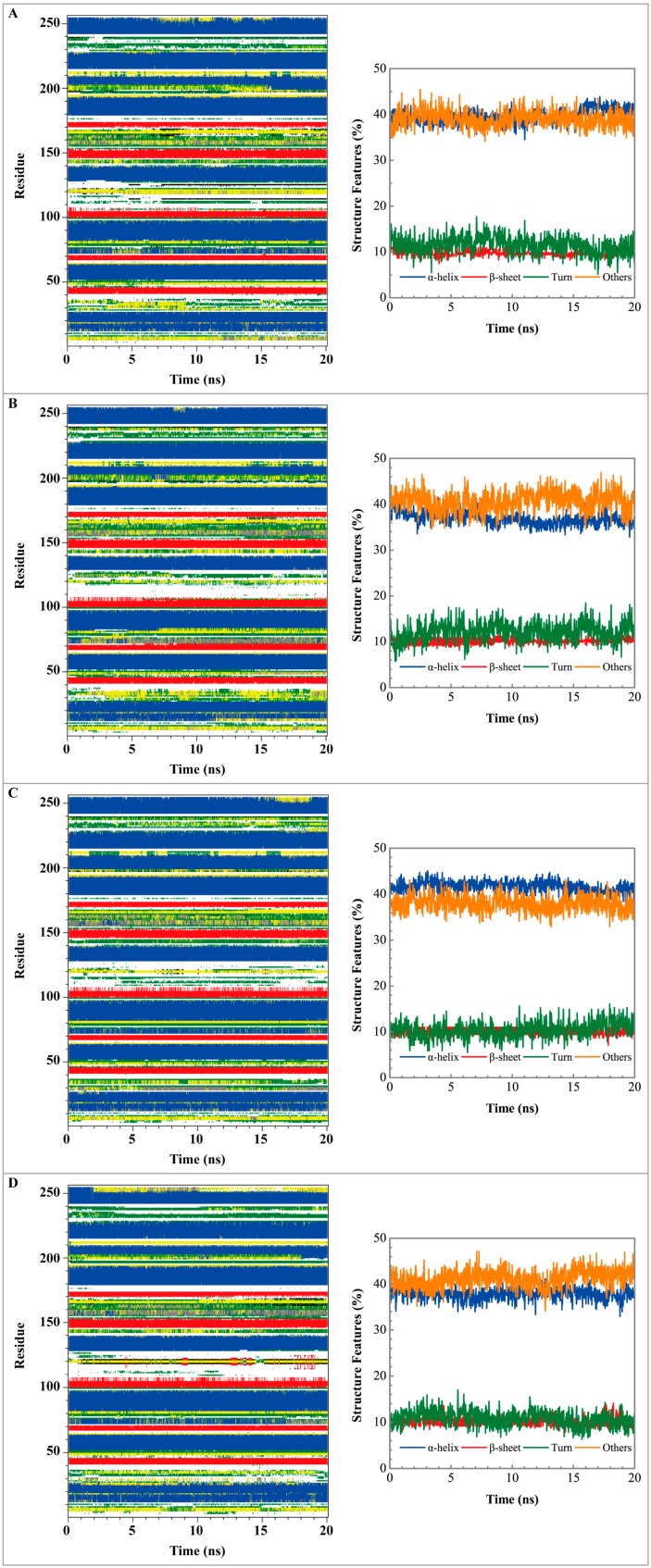
Secondary structure assignment and secondary structural feature ratio variations for paraplegin protein in (**A**) apo form and complexes with (**B**) 5-hydroxy-l-tryptophan; (**C**) saussureamine C; and (**D**) -(2-carboxyphenyl)-4(3*H*)-quinazolinone.

Figure 5 displays the variation of root-mean-square deviations of protein and ligand over 20 ns for paraplegin in the apo form and in complexes with three TCM candidates after the MD simulation. Each system of MD simulation tends to stabilize after 16 ns of MD simulation. However, the ligand RMSD for 5-hydroxy-l-tryptophan has three significant variants during MD simulation (10 ns, 13 ns, 17.5 ns). As there is also no significant variance in the total energies for each paraplegin complexes with three TCM candidates (Figure 6), the binding of each ligand does not cause a significant variance for paraplegin protein. Considering the variation of secondary structure assignment and secondary structural feature ratio for paraplegin in apo form and in complexes with three TCM candidates during MD simulation displayed in Figure 7, the feature ratio of α-helices for paraplegin complexes with 5-hydroxy-l-tryptophan and 3-(2-carboxyphenyl)-4(3*H*)-quinazolinone have slightly decreased while the feature ratio of α-helices for paraplegin complexes with saussureamine C have slightly increased.

**Figure 8:**
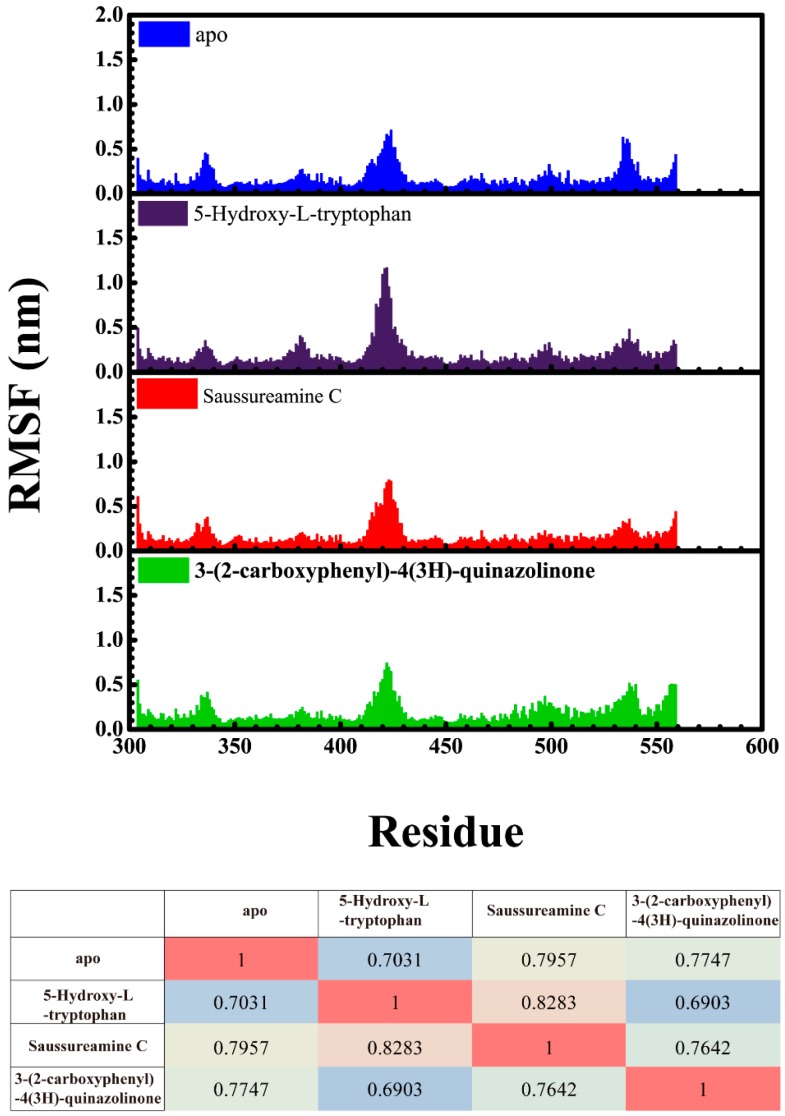
Root mean square fluctuation (RMSF) for residues in apo form of paraplegin protein and in paraplegin complexes with 5-hydroxy-l-tryptophan, saussureamine C, and 3-(2-carboxyphenyl)-4(3*H*)-quinazolinone and the correlation between each complex.

**Figure 9:**
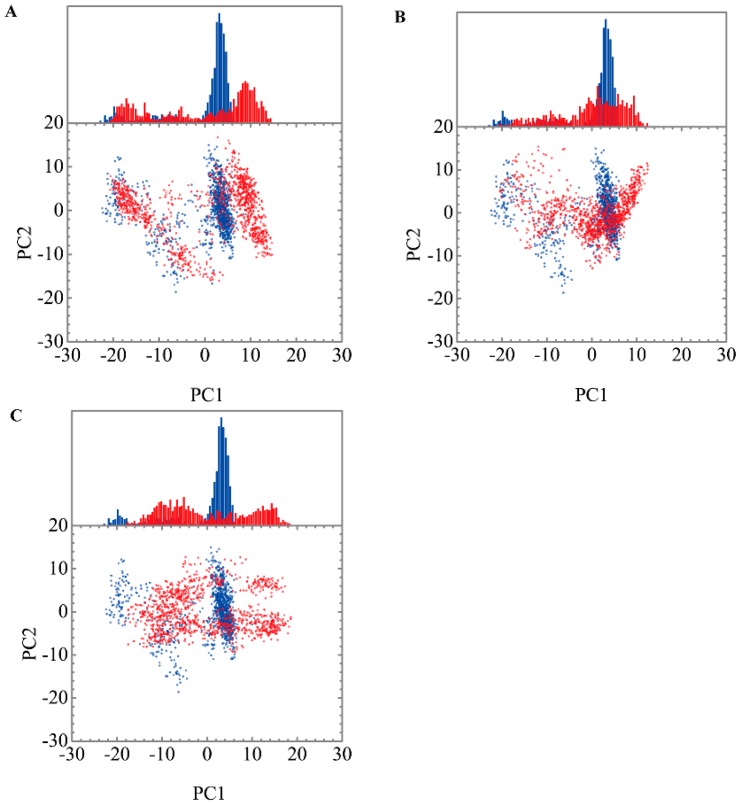
Eigenvector distribution of paraplegin protein in apo form (blue) and paraplegin complexes (red) with (**A**) 5-hydroxy-l-tryptophan; (**B**) saussureamine C; and (**C**) 3-(2-carboxyphenyl)-4(3*H*)-quinazolinone.

Root mean square fluctuations (RMSFs) for each residue in apo form of paraplegin protein and in paraplegin complexes with three TCM candidates over 20 ns MD simulation and the correlation between each complex are shown in Figure 8. The flexibility of residues of paraplegin protein was similar, which illustrated that each ligand does not cause a significant variance for paraplegin protein under dynamic condition after docking. Considering the correlation between each complex, paraplegin complexes with 5-hydroxy-l-tryptophan and saussureamine C have similar variations for paraplegin protein with a correlation index of 0.8283. However, as the correlation index between paraplegin complexes with 5-hydroxy-l-tryptophan and paraplegin in the apo form is only 0.7031, it indicates that 5-hydroxy-l-tryptophan may cause a significant variance for the residues close to the binding domain under dynamic conditions. For paraplegin complexes with 3-(2-carboxyphenyl)-4(3*H*)-quinazolinone, the correlation index with the apo form of paraplegin was better than paraplegin complexes with other two candidates, which indicates that 3-(2-carboxyphenyl)-4(3*H*)-quinazolinone causes a different variation in the residues of paraplegin protein close to the binding domain after MD simulation than the other two candidates. Similar results were also obtained in eigenvector distribution of paraplegin protein complexes shown in Figure 9. 5-hydroxy-l-tryptophan have a significant shift for the PC1 and 3-(2-carboxyphenyl)-4(3*H*)-quinazolinone caused a significant variation in the eigenvector distribution of paraplegin protein.

**Figure 10:**
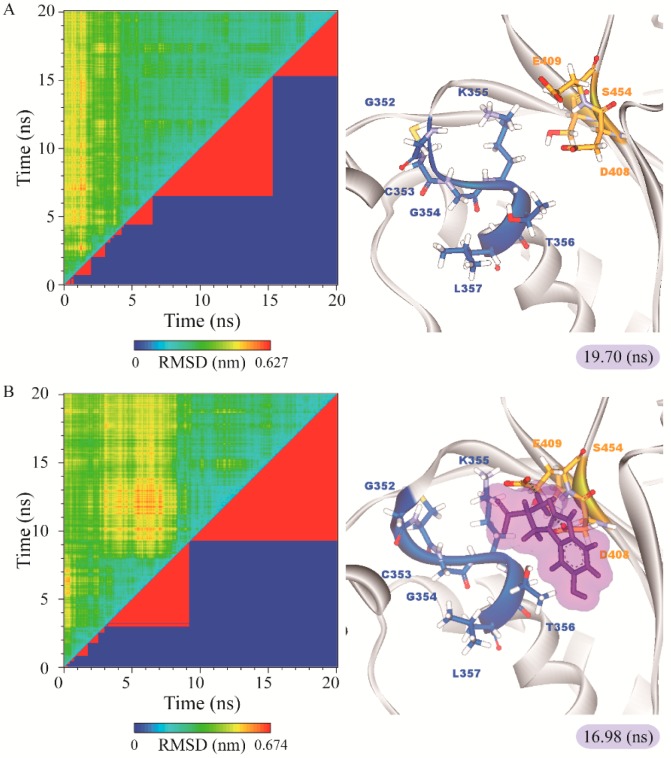

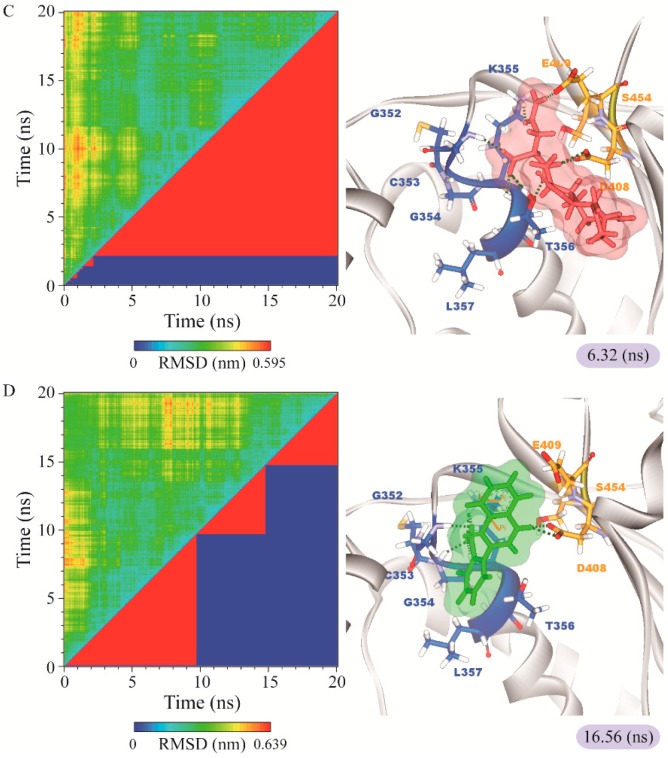
RMSD matrix and clustering diagram of MD comformations with the middle RMSD structure in the major cluster for (**A**) apo form of paraplegin protein and paraplegin complexes with (**B**) 5-hydroxy-l-tryptophan; (**C**) saussureamine C; and (**D**) 3-(2-carboxyphenyl)-4(3H)-quinazolinone and the correlation between each complex.

**Figure 11:**
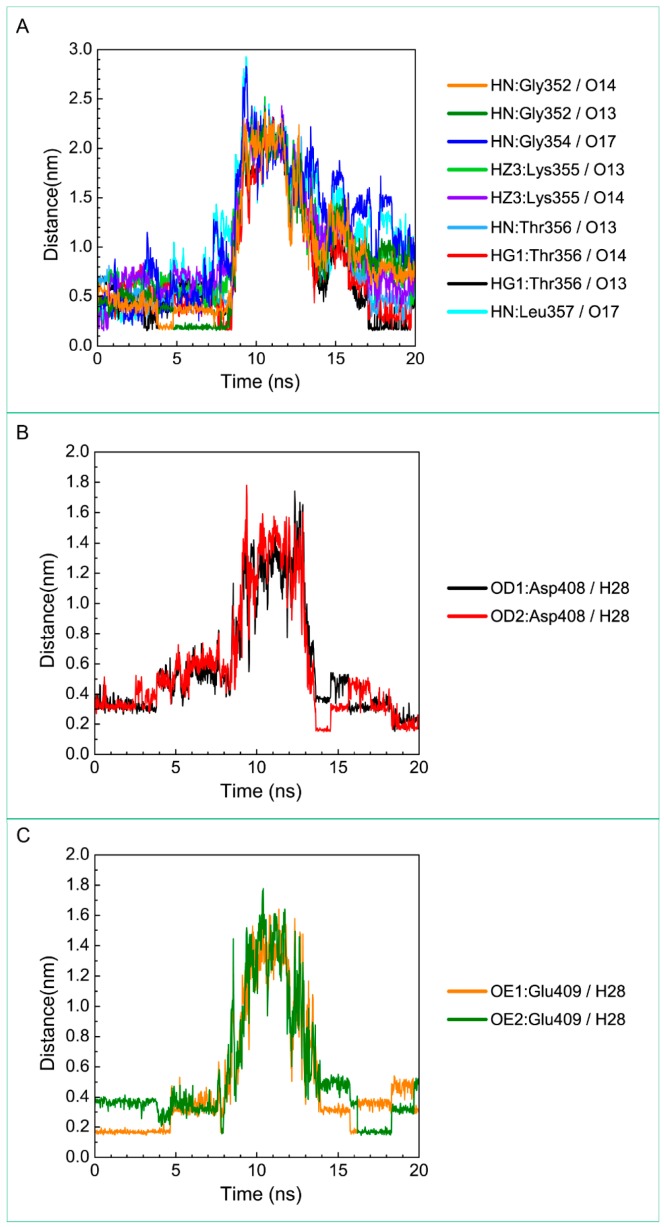
Distance variation of H-bonds for paraplegin complexes with 5-hydroxy-l-tryptophan during MD simulation. Display the distances between compound and amino acids (**A**) Gly352, Gly354, Lys355, Thr356, Leu357, (**B**) Asp408, and (**C**) Glu409.

The representative structures of paraplegin in apo form and in complexes with the three TCM candidates after MD simulation were decided by the RMSD values and graphical depiction of the clusters analysis and displayed in Figure 10. Comparing to the interactions in docking simulation, 5-hydroxy-l-tryptophan cannot binding stably in the binding domain and loses all the H-bonds with key residues in the chain from Gly352 to Thr356 (blue) after MD simulation. To consider the variances of H-bonds during MD simulation, the H-bond occupancy and distance variations during MD simulation are displayed in Table 2 and Figure 11. In Figure 11, all H-bonds have disconnected since 8 ns of MD simulation and connected after 17 ns of MD simulation. It shows that the docking pose of 5-hydroxy-l-tryptophan is not stable under MD simulation.

**Figure 12:**
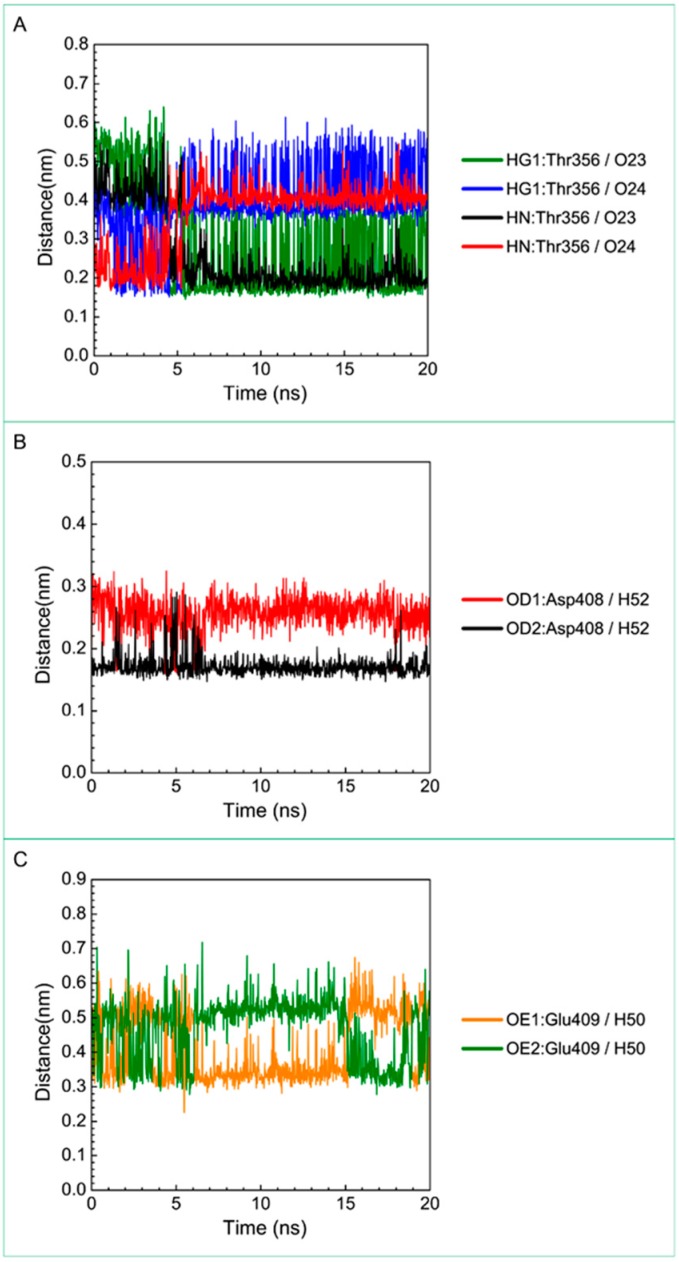
Distance variation of H-bonds for paraplegin complexes with saussureamine C during MD simulation. Display the distances between compound and amino acids (**A**) Thr356; (**B**) Asp408; and (**C**) Glu409.

**Figure 13:**
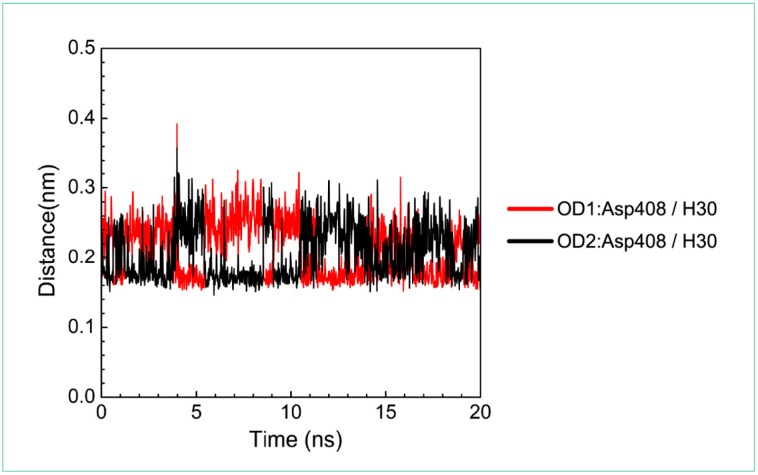
Distance variation of H-bonds for paraplegin complexes with 3-(2-carboxyphenyl)-4(3*H*)-quinazolinone during MD simulation. Display the distances between compound and amino acid Asp408.

For the other candidates, the paraplegin complexes with saussureamine C tends to stable after a short period of MD simulation, and the interactions between protein and ligand have increased to become more stable and paraplegin complexes with 3-(2-carboxyphenyl)-4(3*H*)-quinazolinone is also binding stably during MD simulation. The distance variations of H-bonds displayed in Figure 12 illustrated that the H-bonds have been variated at 5 ns of MD simulation and tend to stabilized. The H-bonds between Thr356 and saussureamine C were shifted between two equal carboxylic ions of saussureamine C due to the rotation (Figure 12A), and the H-bonds between saussureamine C and Glu409 were shifted repeatedly between the equal oxygen atoms OE1 and OE2 of Glu409. In Figure 13, 3-(2-carboxyphenyl)-4(3*H*)-quinazolinone also has stable H-bonds with Asp408, and the H-bonds were shifted repeatedly between equal oxygen atoms OD1 and OD2 of Asp408. Both saussureamine C and 3-(2-carboxyphenyl)-4(3*H*)-quinazolinone remain the interactions with key residues in the chain from Gly352 to Thr356 (blue) and residues Asp408, Glu409, Ser454 (yellow), which indicates that the binding poses of those two TCM candidates in the docking simulation are stable under dynamics condition.

**Figure 14:**
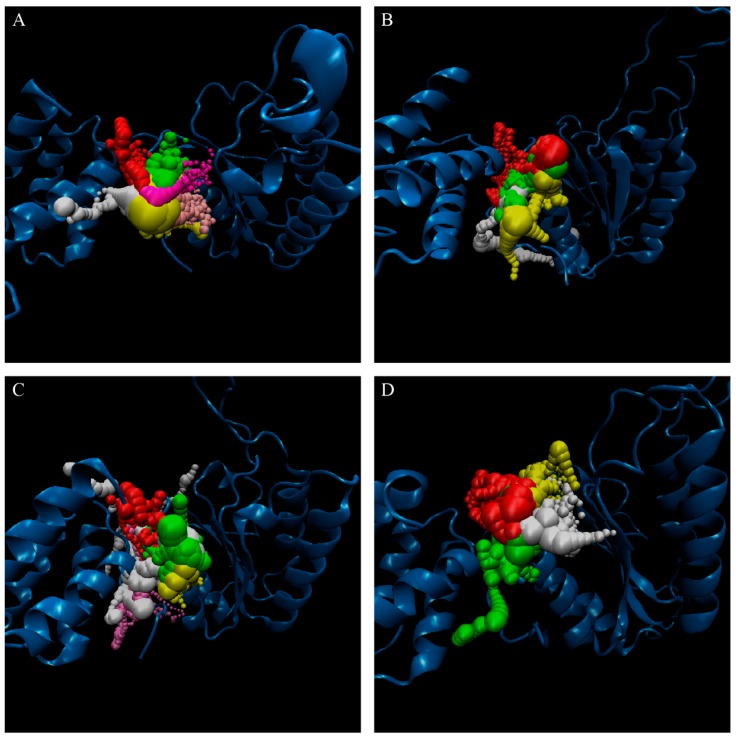
Analysis of transport pathways for paraplegin protein in (**A**) apo form and complexes with (**B**) 5-hydroxy-l-tryptophan; (**C**) saussureamine C; and (**D**) 3-(2-carboxyphenyl)-4(3*H*)-quinazolinone.

**Figure 15:**
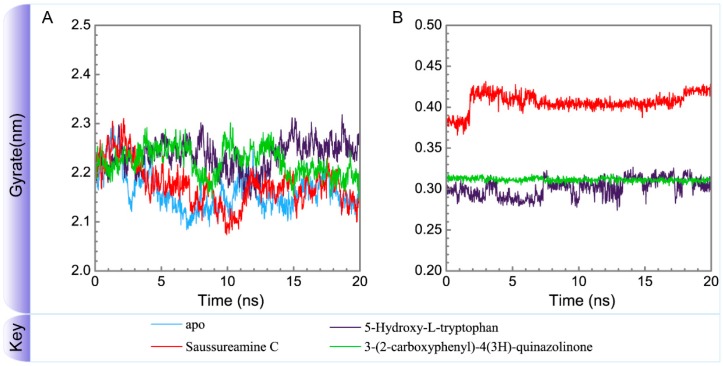
Variation of radii of gyration of (**A**) protein and (**B**) ligand over 20 ns for paraplegin in apo form and in complexes with three TCM candidates.

**Figure 16:**
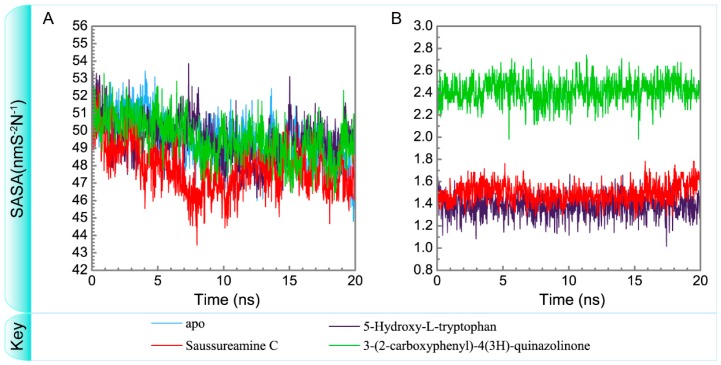
Variation of total solvent accessible surface area of (**A**) protein and (**B**) ligand over 20 ns for paraplegin in apo form and in complexes with three TCM candidates.

**Figure 17:**
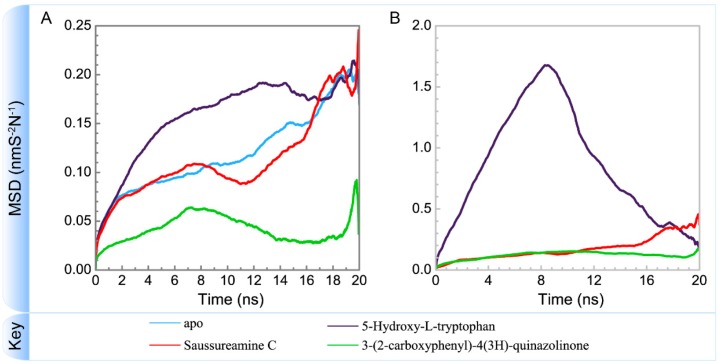
Variation of mean square displacements of (**A**) protein and (**B**) ligand over 20 ns for paraplegin in apo form and in complexes with three TCM candidates.

To consider the effect of each ligand upon the paraplegin protein after docking, Figure 14, Figure 15, Figure 16 and Figure 17 illustrated the transport pathways for paraplegin proteins in each complex and the variation of radii of gyration, total solvent accessible surface area, mean square displacements for paraplegin protein and each ligand during MD simulation, respectively. As the mean square displacements of ligand for 5-hydroxy-l-tryptophan has a sharply variation during MD simulation, it indicates that the docking pose of 5-hydroxy-l-tryptophan has a significant variation under dynamic condition and 5-hydroxy-l-tryptophan may not be binding stably in the binding domain. For the other candidates, the system tends to stable after 3 ns of MD simulation.

The interaction between 5-hydroxy-l-tryptophan and paraplegin protein is not stable enough to retain the 5-hydroxy-l-tryptophan in the binding site under dynamic conditions, so the mean square displacements of 5-hydroxy-l-tryptophan show a sharp variation during MD simulation and the eigenvector distribution of paraplegin protein with 5-hydroxy-l-tryptophan has a significant shift in the PC1 coordinate. As 5-hydroxy-l-tryptophan has left the binding domain during the MD simulation, it causes a significant variance for the residues closed to the binding domain under dynamics condition. These results indicate that 5-hydroxy-l-tryptophan is not a potential candidate for docking with paraplegin.

For saussureamine C, the correlation index between paraplegin complexes with saussureamine C and paraplegin in the apo form is 0.7957, which is better than paraplegin complexes with the other compounds. Saussureamine C has a similar effect as 5-hydroxy-l-tryptophan for paraplegin complexes (the correlation index is 0.8283), but the eigenvector distribution of paraplegin protein with saussureamine C and the docking pose of saussureamine C after MD simulation indicate that eigenvector distribution of saussureamine C does not have a significant shift in the PC1 coordinate and the interactions between saussureamine C and protein have increased to become more stable after MD simulation.

For 3-(2-carboxyphenyl)-4(3*H*)-quinazolinone, although the eigenvector distribution of paraplegin protein with 3-(2-carboxyphenyl)-4(3*H*)-quinazolinone indicates a significant variation in the PC1 coordinate, the docking poses in Figure 10 display that 3-(2-carboxyphenyl)-4(3*H*)-quinazolinone still has interactions with key residues in the chain from Gly352 to Thr356 and residues Asp408, Glu409, Ser454 with a docking pose other than saussureamine C. Both saussureamine C and 3-(2-carboxyphenyl)-4(3*H*)-quinazolinone thus have stable docking poses with paraplegin protein to maintain the compounds in the binding domain.

## 4. Conclusions

This study aimed to identify potential lead drugs from among compounds isolated from TCMs for further development of inhibitors against paraplegin protein for the treatment of coronary artery disease. The top TCM compounds, 5-hydroxy-l-tryptophan, saussureamine C, and 3-(2-carboxyphenyl)-4(3*H*)-quinazolinone, have potential binding affinities and H-bonds with key residues Lys355, Asp408, Glu409, and hydrophobic contacts with residues Pro351, Gly352, Lys355, Thr356, Asp408, and Glu409. However, the interactions between paraplegin protein and 5-hydroxy-l-tryptophan are not stable under dynamic conditions while the other TCM candidates, saussureamine C, and 3-(2-carboxyphenyl)-4(3*H*)-quinazolinone maintain similar docking poses under dynamic conditions. In addition, those two TCM compounds retain the interactions with key residues in the chain from Gly352 to Thr356 and residues Asp408, Glu409, Ser454, which stabilize their docking poses in the binding site under dynamic conditions. Hence, we propose these two TCM compounds, saussureamine C and 3-(2-carboxyphenyl)-4(3*H*)-quinazolinone, as potential lead drug candidates isolated from TCMs for further study in the drug development process with paraplegin protein for coronary artery disease.

## Figures and Tables

**Table 1:** Scoring functions of top candidates from TCM database screening and correlated residues of paraplegin for H-bonds.

Name	Dock Score	H-Bond Forming Residues	H-Bond Quantity
5-Hydroxy-l-tryptophan	203.573	Lys355, Leu357, Asp408, Glu409	**4**
Saussureamine C	188.993	Lys355, Thr356, Asp408, Glu409, Ser454, Asn456	**6**
3-(2-Carboxyphenyl)-4(3*H*)-quinazolinone	180.181	Gly352, Lys355, Asp408, Glu409	**4**
Saussureamine B	164.449	Lys355, Thr356	**2**
Crotalaburnine	160.377	Asp408, Glu409	**2**
Labiatic acid	154.729	Gly354, Lys360, His492	**3**
Saussureamine A	152.977	Gly352, Lys355, Asp408	**3**
l-Valine-l-valine anhydride	149.351	Gly352, Asp408, Glu409	**3**
*N*-Methyl tyramine-*O*-alpha-l-rhamnopyranoside	147.058	Gly354, Asp408, Glu409	**3**
Riddelline	146.166	Gly352, Asp408, Glu409	**3**

**Table 2:** Analysis of H-bond occupancies for each ligand during MD simulation.

Name	H-Bond Interaction	Occupancy
5-Hydroxy-l-tryptophan	Gly352: HN/O13	19.00%
	Gly352: HN/O14	12.80%
	Thr356: HG1/O13	18.00%
	Thr356: HG1/O14	13.30%
	Glu377: OE2/H28	10.00%
	Asp408: OD1/H28	36.60%
	Asp408: OD2/H28	38.00%
	Glu409: OE1/H28	51.50%
	Glu409: OE2/H28	37.10%
Saussureamine C	Gly352: HN/O23	30.77%
	Gly352: HN/O24	69.83%
	Thr356: HG1/O23	62.74%
	Thr356: HG1/O24	15.48%
	Thr356: HN/O23	75.72%
	Thr356:HN/O24	23.98%
	Thr356: OG1/H52	61.64%
	Asp408: OD1/H52	100.00%
	Asp408: OD2/H52	100.00%
	Glu409: OE1/H50	46.15%
	Glu409: OE2/H50	19.78%
	Ser454: HG1/O28	76.02%
	Asn456: HD22/N27	99.70%
	Asn456: OD1/H50	100.00%
3-(2-carboxyphenyl)-4(3*H*)-quinazolinone	Gly352: HN/O11	95.50%
	Gly352: HN/O19	81.70%
	Gly352: HN/O20	88.90%
	Cys353: HN/O19	34.40%
	Cys353: HN/O20	97.10%
	Gly354: HN/O20	96.60%
	Lys355: HZ3/O19	85.70%
	Lys355: HZ3/O20	52.40%
	Lys355: HN/O20	99.40%
	Thr356: HN/O20	51.30%
	Asp408: OD1/H30	100.00%
	Asp408: OD2/H30	100.00%

Cut-off = 0.35 nm.
